# Investigating microbial size classes associated with the transmission of stony coral tissue loss disease (SCTLD)

**DOI:** 10.7717/peerj.15836

**Published:** 2023-08-23

**Authors:** James S. Evans, Valerie J. Paul, Blake Ushijima, Kelly A. Pitts, Christina A. Kellogg

**Affiliations:** 1 St. Petersburg Coastal and Marine Science Center, U.S. Geological Survey, St. Petersburg, Florida, United States of America; 2 Smithsonian Marine Station, Ft. Pierce, Florida, United States of America; 3 Department of Biology & Marine Biology, University of North Carolina at Wilmington, Wilmington, North Carolina, United States of America

**Keywords:** Coral, 16S, Disease, Microbes, Bacteria, SCTLD, Water-borne, Transmission

## Abstract

Effective treatment and prevention of any disease necessitates knowledge of the causative agent, yet the causative agents of most coral diseases remain unknown, in part due to the difficulty of distinguishing the pathogenic microbe(s) among the complex microbial backdrop of coral hosts. Stony coral tissue loss disease (SCTLD) is a particularly destructive disease of unknown etiology, capable of transmitting through the water column and killing entire colonies within a matter of weeks. Here we used a previously described method to (i) isolate diseased and apparently healthy coral colonies within individual mesocosms containing filtered seawater with low microbial background levels; (ii) incubate for several days to enrich the water with coral-shed microbes; (iii) use tangential-flow filtration to concentrate the microbial community in the mesocosm water; and then (iv) filter the resulting concentrate through a sequential series of different pore-sized filters. To investigate the size class of microorganism(s) associated with SCTLD transmission, we used 0.8 µm pore size filters to capture microeukaryotes and expelled zooxanthellae, 0.22 µm pore size filters to capture bacteria and large viruses, and 0.025 µm pore size filters to capture smaller viruses. In an attempt to further refine which size fraction(s) contained the transmissible element of SCTLD, we then applied these filters to healthy “receiver” coral fragments and monitored them for the onset of SCTLD signs over three separate experimental runs. However, several factors outside of our control confounded the transmission results, rendering them inconclusive. As the bulk of prior studies of SCTLD in coral tissues have primarily investigated the associated bacterial community, we chose to characterize the prokaryotic community associated with all mesocosm 0.22 µm pore size filters using Illumina sequencing of the V4 region of the 16S rRNA gene. We identified overlaps with prior SCTLD studies, including the presence of numerous previously identified SCTLD bioindicators within our mesocosms. The identification in our mesocosms of specific bacterial amplicon sequence variants that also appear across prior studies spanning different collection years, geographic regions, source material, and coral species, suggests that bacteria may play some role in the disease.

## Introduction

Coral reefs are estimated to cover less than 1% of the Earth’s surface, yet they support a disproportionately large number of the world’s marine species (Reaka-Kudla, 1997). These “rainforests of the sea” provide critical three-dimensional structures that sustain diverse communities of marine organisms, while also supporting tourism (Spalding et al., 2017) and fishery industries (Grafeld et al., 2017), protecting coastlines from storms (Ferrario et al., 2014), and providing numerous other ecological and economic services, all with an estimated global annual value of $9.9 trillion USD (Costanza et al., 2014). However, coral reefs are facing a pronounced decline worldwide, driven primarily by global climate change, ocean acidification, water pollution, and, increasingly, coral diseases (Pandolfi et al., 2003; Bourne et al., 2009; Weil & Rogers, 2011; Hughes et al., 2017).

Despite playing a substantial role in the degradation of the world’s reefs, our understanding of coral diseases is disproportionately limited, such that to date the causative agents have been conclusively identified for just a handful of coral diseases (Richardson, 1998; Sutherland, Porter & Torres, 2004; Rosenberg et al., 2007; Vega Thurber et al., 2020). Part of this disparity is due to the difficulty of fulfilling Koch’s postulates, which remains the guideline for establishing causation for a disease’s potential etiological agent and includes a complex series of steps involving isolation of the pathogen, culturing, infection of a healthy individual, and re-isolation of the same pathogen from the newly infected individual. In particular, culturing of the causative agent can prove problematic, as most microorganisms are not able to be grown in pure cultures using current techniques. Coral diseases may be especially difficult to investigate, as coral microbiomes are naturally highly diverse and complex (Huggett & Apprill, 2019) and may vary between coral species (Rohwer et al., 2002), conspecific coral colonies (Glasl et al., 2019), geographic regions (Littman et al., 2009), coral holobiont compartments (Sweet, Croquer & Bythell, 2011; Apprill, Weber & Santoro, 2016), across habitats (Pantos et al., 2015; Ziegler et al., 2019; Camp et al., 2020), and through time (Williams et al., 2015), all of which may complicate coral pathogen identification efforts. Additionally, seawater hosts its own diverse microbial communities, making contamination between the coral organism and its external environment difficult to discern (Richardson, 1998), and pathogenic microbes are sometimes identified within the microbiomes of apparently healthy corals (Bourne & Munn, 2005), underscoring the difficulty in distinguishing between beneficial and detrimental microbiome constituents. Further, the route of infection is unknown for most coral diseases (Richardson, 1998), and while some pathogens may be externally sourced, others may be microorganisms that normally exist in a commensal or even mutualistic relationship with their host, suddenly transitioning to a pathogenic state in response to some environmental stimuli (Vega Thurber et al., 2020). Indeed, even when microorganisms are detected in association with disease signs in corals, it can be unclear whether these represent an initial, primary infection, or a secondary, opportunistic infection (Lesser et al., 2007). Given this abundance of complicating factors, it is perhaps no surprise that the agents responsible for most coral diseases remain unidentified.

In 2014, a new coral disease known as stony coral tissue loss disease (SCTLD) emerged off the coast of Miami (Precht et al., 2016), and has since spread throughout the entirety of Florida’s Coral Reef (Muller et al., 2020; Dobbelaere et al., 2022) and much of the wider Caribbean (Alvarez-Filip et al., 2019; Weil et al., 2019; Brandt et al., 2021; Dahlgren et al., 2021; Heres et al., 2021) to become arguably the worst coral epidemic in recorded history. SCTLD is characterized by focal or multifocal tissue loss lesions ranging from subacute to acute that form and progress rapidly (multiple cm day^−1^) (National Oceanic and Atmospheric Administration (NOAA), 2018). The first appearance of gross disease signs can be followed by full mortality of the infected colony on the scale of weeks to months for some of the most highly susceptible species (National Oceanic and Atmospheric Administration (NOAA), 2018). More than 20 species are known to be susceptible to SCTLD, including important reef-builders and several species currently listed under the U.S. Endangered Species Act (National Oceanic and Atmospheric Administration (NOAA), 2018), with some of the most highly susceptible species in the most heavily impacted regions losing more than 97% of their pre-SCTLD colonies (Precht et al., 2016). It has also recently been suggested that marine vessel traffic may potentially contribute to the spread of this disease (Dahlgren et al., 2021; Rosenau et al., 2021; Evans, Paul & Kellogg, 2022; Studivan et al., 2022a), underscoring the time-sensitive nature of SCTLD research, as some regions remain unaffected.

The transmission of SCTLD *ex situ* (Aeby et al., 2019) and the contagious nature of the disease (Muller et al., 2020) imply a biological agent, and the efficacy of various antibiotics (Aeby et al., 2019; Neely et al., 2020, 2021; Shilling, Combs & Voss, 2021; Walker et al., 2021) in treating SCTLD lesions suggests possible bacterial involvement. Indeed, investigations into bacterial communities associated with SCTLD have begun to identify consistent bacterial taxa and even 100% identical amplicon sequence variants (ASVs) associated with the disease (Meyer et al., 2019; Rosales et al., 2020; Becker et al., 2021; Clark et al., 2021; Evans, Paul & Kellogg, 2022; Evans et al., 2022; Huntley et al., 2022; Studivan et al., 2022b). Other evidence, however, such as the lack of observable bacteria in light microscopy and transmission electron microscopy examinations of lesions (Landsberg et al., 2020; Work et al., 2021), and the observation of viral-like particles in transmission electron microscopy (Work et al., 2021) suggests SCTLD causation may potentially involve viruses. Ciliates (microeukaryotes) have also been detected in greater abundance in SCTLD lesions compared to healthy or unaffected coral tissue (Rosales et al., 2022). Given that different microbial groups may require very different treatments and prevention strategies, determining whether the disease is caused by microeukaryotes, bacteria, viruses, chemicals or toxins, or some combination of these agents, is a critical next step towards mitigating and preventing the spread of this deadly disease.

It has been established that SCTLD exhibits waterborne transmission (Aeby et al., 2019; Muller et al., 2020), indicating that infected corals must shed the causative agent into the surrounding water column. By screening this water, it may be possible to eliminate some of the background microorganisms otherwise associated with the coral host and more quickly identify possible pathogens (Evans et al., 2022). The use of sterilized water as the base for this “shedding” would further serve to exclude additional “background noise” from consideration. Here we use the methodology described by Evans et al. (2022), employing closed-system mesocosms containing UV-treated, filtered seawater and either an apparently healthy coral colony or one infected with SCTLD; tangential flow filtration to concentrate the mesocosm microbial community and account for SCTLD’s unknown infectious dose; and sequential size fractionation to isolate different components of the mesocosm microbial community. We further attempt to identify the size of SCTLD’s causative agent by applying these different size fractionation filters directly to healthy corals and monitoring for the onset of SCTLD symptoms. Finally, we characterize the microbial community associated with the 0.22 µm pore size mesocosm filters.

## Materials and Methods

Three iterations of this experiment were performed: one in October 2019, one in November 2020, and one in March 2021, hereafter referred to as Run 1, Run 2, and Run 3, respectively. All three runs generally followed the methodology described by Evans et al. (2022), with the addition of disease transmission tests, as described below. Each run varied slightly from one another based on knowledge acquired during preceding runs; all variations are likewise detailed below. Corals used in these experiments were collected under Florida Keys National Marine Sanctuary permits FKNMS-2017-128-A2 and FKNMS-2019-160; additional details related to these corals are outlined in Table 1. Research conducted under this project was performed under the CRF NOAA FKNMS permit FKNMS-2019-012.

**Table 1:** Outcomes of transmission experiments by experimental run and mesocosm type (diseased or healthy).

Experimental run	Diseased donor corals									Healthy donor corals								
	Donor coral				Treatment + Transmission outcome					Donor coral				Treatment + Transmission outcome				
	ID	Species	VcpA test	Source location	0.8 μm filter	0.22 μm filter	0.025 μm filter	0.22 μm + 0.025 μm filters	TFF filtrate	ID	Species	VcpA test	Source location	0.8 μm filter	0.22 μm filter	0.025 μm filter	0.22 μm + 0.025 μm filters	TFF filtrate
Run 1 (October 2019)	Cnat15	CNAT	–	FL Keys	ND	–	–	–	ND	CnH-101	CNAT	–	KW Nursery, FL	ND	–	–	–	ND
	Cnat-20	CNAT	–	FL Keys	ND	–	–	–	ND	CnH-104	CNAT	–	KW Nursery, FL	ND	–	–	–	ND
	Cnat-23	CNAT	–	FL Keys	ND	–	–	–	ND	CnH-106	CNAT	–	KW Nursery, FL	ND	–	–	–	ND
	Mcav8	MCAV	+	FL Keys	ND	–	–	–	ND	McH-101	MCAV	–	FL Keys	ND	–	–	–	ND
	Mcav-17	MCAV	+	FL Keys	ND	X	–	–	ND	McH-103(4)	MCAV	–	KW Nursery, FL	ND	–	–	–	ND
	Mcav-18	MCAV	+	FL Keys	ND	X	–	–	ND	McH-104	MCAV	–	KW Nursery, FL	ND	–	–	–	ND
	Ofav-9	OFAV	–	FL Keys	ND	–	–	–	ND	OfH-100	OFAV	–	KW Nursery, FL	ND	–	–	–	ND
	Ofav-16	OFAV	+	FL Keys	ND	–	–	–	ND	OfH-101	OFAV	–	KW Nursery, FL	ND	–	–	–	ND
	Ofav-19	OFAV	–	FL Keys	ND	–	–	–	ND	OfH-104	OFAV	–	KW Nursery, FL	ND	–	–	–	ND
	Ofav-26	OFAV	–	FL Keys	ND	–	–	–	ND	OfH-105(3)	OFAV	–	Ft. Lauderdale, FL	ND	–	–	–	ND
Run 2 (November 2020)	CnD-7	CNAT	–	Marathon, FL	–	–	–	–	–	CnH-104 [Cnat-M1]	CNAT	–	KW Nursery, FL	–	–	–	–	–
	CnD-8	CNAT	–	Marathon, FL	–	–	–	–	–	CnH-101 [Cnat-M2]	CNAT	–	KW Nursery, FL	–	–	–	–	–
	CnD-9	CNAT	–	Marathon, FL	–	–	–	–	–	CnH-no# [Cnat-M3]	CNAT	–	KW Nursery, FL	–	–	–	–	–
	DlD-1	DLAB	–	Marathon, FL	–	–	–	–	–	Dlab-2 & 3	DLAB	–	Dry Tortugas	–	–	–	–	–
	McD-56	MCAV	–	Marathon, FL	–	–	–	–	–	Mcav-2SQ	MCAV	–	Dry Tortugas	–	–	–	–	–
	McD-57	MCAV	–	Marathon, FL	–	–	–	–	–	McH-103(4) [Mcav-M1]	MCAV	–	KW Nursery, FL	–	–	–	–	–
	McD-58	MCAV	–	Marathon, FL	–	–	–	–	–	McH-101 [Mcav-M2]	MCAV	–	FL Keys	–	–	–	–	–
	FtL McD-30-31	MCAV	–	Broward, FL	–	–	–	–	–	McH-102 [Mcav-M3]	MCAV	–	KW Nursery, FL	–	–	–	–	–
	OaD-1	OANN	–	Marathon, FL	–	–	–	–	–	OfH-108 [Ofav-M1]	OFAV	–	KW Nursery, FL	–	–	–	–	–
	OaD-2	OANN	–	Marathon, FL	–	–	–	–	–	OfH-106 [Ofav-M2]	OFAV	–	KW Nursery, FL	–	–	–	–	–
Run 3 (March 2021)	CnD-16	CNAT	–	Vaca Key, FL	X	–	–	ND	X	Cnat-8-4	CNAT	–	Dry Tortugas	X	–	X	ND	–
	CnD-17	CNAT	–	Vaca Key, FL	–	–	–	ND	X	CnH-100	CNAT	–	KW Nursery, FL	X	–	–	ND	–
	CnD-18	CNAT	–	Vaca Key, FL	–	–	–	ND	X	CnH-101	CNAT	–	KW Nursery, FL	–	–	–	ND	X
	CnD-19	CNAT	–	Vaca Key, FL	–	–	–	ND	–	BB-Dl-1	DLAB	–	Biscayne Bay, FL	–	–	–	ND	X
	CnD-20	CNAT	–	Vaca Key, FL	–	X	–	ND	–	BB-Dl-2	DLAB	–	Biscayne Bay, FL	–	–	X	ND	X
	CnD-21	CNAT	–	Vaca Key, FL	–	–	–	ND	–	Dlab-3	DLAB	–	Dry Tortugas	X	–	–	ND	X
	CnD-22	CNAT	–	Vaca Key, FL	–	–	–	ND	–	BB-Ps-1	PSTR	–	Biscayne Bay, FL	–	–	–	ND	–
	CnD-23	CNAT	–	Vaca Key, FL	–	X	–	ND	–	Pstr-4	PSTR	–	Dry Tortugas	–	–	–	ND	–
	CnD-24	CNAT	–	Vaca Key, FL	ND	–	–	ND	–	Pstr-5	PSTR	–	Dry Tortugas	X	–	–	ND	–
	CnD-25	CNAT	ND	Vaca Key, FL	–	–	–	–	X	Pstr-10	PSTR	–	Dry Tortugas	X	–	–	ND	X
	PsD-5	PSTR	–	Vaca Key, FL	–	X	–	ND	–									

**Note:**

“Donor Coral” refers to the corals used in mesocosms as “donors,” or sources of microbes, with sample ID, species, VcpA (*Vibrio corallilyticus*) test outcome, and original source location for each donor coral indicated. CNAT, *Colpophyllia natans*; DLAB, *Diploria labyrinthiformis*; MCAV, *Montastraea cavernosa*; OANN, *Orbicella annularis*; OFAV, *Orbicella faveolata*; PSTR, *Pseudodiploria strigosa*. Sample ID names in brackets indicate the experimental ID used during a run, if different from the coral’s long-term ID. A VcpA test result of “+” indicates the colony was positive for *V.corallilyticus* and a “−” indicates a negative result. For location, “FL” = Florida and “KW” = Key West. The SCTLD transmission outcome for each receiver coral (an X indicates SCTLD transmission (*i.e*., tissue loss in the receiver coral) and “−” indicates no observed disease transmission) for each treatment type (0.8, 0.22, 0.025, and 0.22 μm + 0.025 μm pore size filters, and tangential flow filtration (TFF) liquid filtrate (<100 kDa)) sourced from each donor coral is indicated. “ND” (“not Determined”) indicates a particular test or treatment was not performed.

### Coral collection and mesocosm creation

Between April 2018 and September 2020, fragments and whole colonies of apparently healthy corals were collected from Florida reefs or coral nurseries ahead of the disease front (locations detailed in Table 1) and transported by cooler or bucket back to shore for use in Runs 1, 2, and/or 3. Colonies were then wrapped in seawater-moistened bubble wrap and transported, *via* cooler, to the Smithsonian Marine Station (SMS) in Fort Pierce, FL, USA. Upon arrival at SMS, apparently healthy colonies were transferred into indoor, temperature-controlled (~25.5 °C) water tables containing UV-treated and filtered seawater (FSW). This FSW was originally collected ~1,600 m offshore and processed through a series of filters, UV-treated *via* a Coralife Turbo-Twist 12× UV Sterilizer with a 36 W UVC lamp and a 254 nm radiation wavelength, and passed through a 0.22 µm pore size polyethersulfone membrane filter to reduce the microbial load. These apparently healthy corals were maintained within these systems, along with numerous other colonies, as part of the SMS long-term healthy coral stock. At the start of each experimental run, all healthy corals used in this study had been residing within these systems for a minimum of 8 months and were returned to these systems following each run (some donor corals were reused in subsequent runs, as indicated by repeated sample IDs in Table 1). Additional details on these facilities, the FSW system, and the husbandry of these corals are described by Ushijima et al. (2020).

Immediately prior to each experimental run, fragments of SCTLD-symptomatic corals were collected by divers from Florida reefs (see Table 1 for locations) and transported to SMS in the same manner as the healthy corals. All diseased fragments collected exhibited visual signs consistent with the SCTLD case definition (National Oceanic and Atmospheric Administration (NOAA), 2018). Upon arrival at SMS, fouling epibionts were removed and a VcpA *RapidTest* (mAbDx, Inc., Eugene, OR, USA) was performed for each diseased donor coral to test for the presence of *Vibrio coralliilyticus*, as described by Ushijima et al. (2020). The VcpA *RapidTest* results for all donor corals are shown in Table 1. For all three runs, diseased corals were then transferred directly into individual mesocosm buckets containing ~18 L of the UV-treated FSW and a weighted airline for water circulation and oxygenation. At the same time, apparently healthy corals were transferred from their indoor housing facilities into identical individual mesocosm buckets. The selected healthy colonies were chosen to match as closely as possible to the size and species of diseased corals that had been collected; however, due to the opportunistic collection of available diseased corals, the closest corresponding healthy match was sometimes of the same genus or family (Table 1). During each run, all individual mesocosm buckets were collectively housed within outdoor water tables containing recirculating freshwater maintained at ~28 °C and located under a mesh canopy to allow some sunlight attenuation. Separate “healthy” and “diseased” water tables were maintained to prevent cross-contamination between the different mesocosm types. During each run, all corals were incubated within these mesocosms for 2–5 days to enrich the water with microbes.

### Microbial community concentration

Following the incubation period, each coral colony was removed from its mesocosm, and the remaining seawater prefiltered through an ethanol-sterilized 200 µm (Run 1) or 106 µm (Runs 2 & 3) pore size mesh screen to remove any particulates. Using a peristaltic pump, the water from each mesocosm was then pumped through a tangential flow filtration (TFF) manifold (Pall corporation; Port Washington, New York, USA) containing five Centramate 100 kDa filter cassettes, as described by Evans et al. (2022). Briefly, anything larger than 100 kDa (including microeukaryotes, bacteria, and viruses) is retained within the sample, while anything smaller than 100 kDa passes out of the system as filtrate (Paul, Jiang & Rose, 1991). Through this process, the microbial community is substantially concentrated while the overall working volume is reduced; here, each mesocosm microbial community was concentrated down from ~18 L to ≤350 mL final volume. A sample (~18 L) of the UV-treated FSW used as a base for the mesocosms was also processed through the TFF system to assess the baseline mesocosm microbial load during Run 3. The TFF filtrate from each sample was also retained for use as a treatment in the transmission experiment (Runs 2 & 3), as described below. For all three experimental runs, after concentrating each mesocosm sample the system was flushed with reverse osmosis (RO) water for 2 min to remove or dilute any residual microbes prior to processing the next mesocosm. All healthy mesocosms were processed first, then the TFF system was flushed with bleach solution (40 mL of ∼5% bleach in 5 L deionized or reverse osmosis water) for 1 h and held overnight while permeated with bleach. The next day, the system was flushed with reverse osmosis (RO) water for 2 min prior to processing diseased mesocosms to prevent cross-contamination between mesocosm types. Following the conclusion of each experimental run, the TFF system was flushed with bleach solution for 1 h, RO water for 2 min, and a 0.1 N NaOH solution for 5 min, then disassembled and stored. Between experimental runs, the filter cassettes were stored at 4 °C fully submerged in 0.1 N NaOH solution. At the start of an experimental run, the system was reassembled and flushed with deionized water for 30 min to purge the NaOH storage agent from the filter cassettes. Additional details on this process are described by Evans et al. (2022).

### Microbial community size fractionation

**Figure 1:**
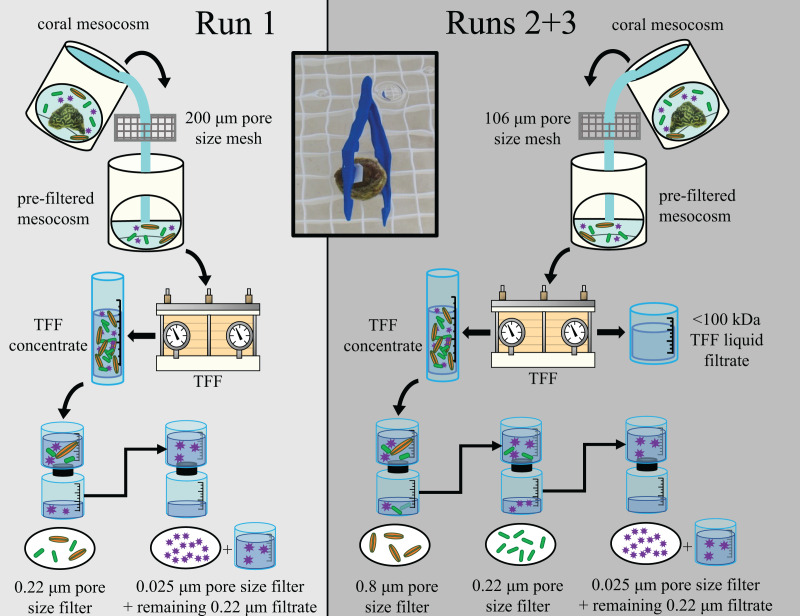
Diagram outlining experimental design for the three experimental runs (Runs 1, 2, and 3). Figure adapted from Evans et al. (2022). The experimental design for Run 1 is shown in the left (light gray) and for Runs 2 & 3 in the right (dark gray). Water from coral mesocosms was first filtered through a 106 or 200 μm pore size mesh screen, then concentrated *via* tangential flow filtration (“TFF”). The resulting concentrated microbial community (“TFF concentrate”) was then sequentially filtered to size fraction different sized components of the mesocosm microbial community. For Run 1, the TFF concentrate was first passed through a 0.22 μm pore size filter to capture larger microorganisms such as microeukaryotes (orange spindles) and bacteria (green rods). For Runs 2 & 3, the TFF concentrate was first passed through a 0.8 μm pore size filter to retain the microeukaryote-sized fraction separately, and the resulting filtrate was then passed through a 0.22 μm pore size filter to retain the bacteria-sized fraction separately. The liquid filtrate resulting from the TFF process (“<100 kDa TFF liquid filtrate”) was also retained as an additional treatment to account for chemicals/toxins/toxicants <100 kDa. For all three runs, the filtrate resulting from the 0.22 μm pore size filtration was subsequently filtered through a 0.025 μm pore size filter to capture most viruses (purple stars), and any liquid filtrate remaining unfiltered after 5 h (“remaining 0.22 μm filtrate”) was also retained and applied to healthy “receiver” corals along with the corresponding 0.025 μm pore size filter during the transmission experiments. The inset photo demonstrates how filters were applied to receiver corals for the transmission experiments. Photos by James Evans and Christina Kellogg.

To partition the microbial community present within each mesocosm’s TFF concentrate into different microbial size classes, each concentrate (≤350 mL) was sequentially filtered through different pore size filters. For Run 1, the TFF concentrate was first passed through a sterile filter unit containing a ~47 mm diameter, 0.22 µm pore size nitrocellulose membrane to capture the larger portion of the mesocosm microbial communities, including microeukaryotes, expelled Symbiodiniaceae (zooxanthellae), and bacteria. The filtrate from the 0.22 µm filtration was then passed through an autoclave-sterilized 47 mm diameter, 0.025 µm pore size mixed ester filter to retain the viral-sized component. Based on the transmission results of Run 1, for Runs 2 and 3, the larger size fraction was further parsed apart by first passing the TFF concentrate through a sterile filter unit containing a 50 mm diameter, 0.8 µm pore size cellulose nitrate membrane to capture primarily the microeukaryotes and expelled Symbiodiniaceae. The resulting filtrate was then passed sequentially through a ~47 mm diameter, 0.22 µm pore size nitrocellulose membrane to capture primarily bacteria, and then an autoclave-sterilized 47 mm diameter, 0.025 µm pore size mixed ester filter to retain the viral-sized component. During all three runs, filtration through the 0.025 µm pore size filter was slow, presumably due to the large number of viral particles present within the concentrated microbial communities, and was consequently stopped after ~5 h due to time constraints. Any unfiltered virus fraction remaining after ~5 h (0–300 mL; actual volumes per sample available in Kellogg, Evans & Voelschow, 2023) was retained along with the filter; this occurred for ~90% of all samples. For all three runs, filters were aseptically cut from the filter units and halved using an ethanol-sterilized razor blade, with ¼ – ½ of each filter retained for the transmission experiment, and the remainder stored at −20 °C for later processing. This process is summarized in Fig. 1, and additional details on the size fractionation process are described by Evans et al. (2022).

### Filter application to healthy corals

To assess the potential contribution of each size class to the transmission of SCTLD, apparently healthy plugs of *Orbicella faveolata* measuring ~3–5 cm in diameter were first acquired from the Coral Restoration Foundation (CRF; Runs 1 & 2) and Mote Marine Laboratory (MML; Run 3) ~2–3 weeks ahead of each experimental run and maintained *ex situ* at SMS. The tanks housing the coral plugs at MML experienced a tissue loss event shortly before the start of Run 3, and consequently individuals with no tissue loss signs (*i.e*., apparently healthy) were specifically selected for use for that run. During all three experimental runs, receiver corals were housed within individual covered plastic aquaria containing ~5 L of UV-treated FSW and an airline. These aquaria were housed within the same outdoor water table setup described above. In all runs, portions of filters were physically applied to these “receiver” corals and held in place using single-use sterile plastic forceps, while any excess liquid virus fraction treatments were poured directly into the water with the receiver coral and airline. In total, the following transmission treatments were trialed: 0.8 µm pore size filters (Runs 2 & 3), 0.22 µm pore size filters (Runs 1, 2, & 3), 0.025 µm pore size filters (Runs 1, 2, & 3), combined 0.22 and 0.025 µm pore size filters (*i.e*., testing for any combination effect of bacterial and viral-sized fractions; Runs 1 & 2), and the liquid TFF filtrate (*i.e*., testing effects of any chemicals/toxins/toxicants present in the mesocosms, Runs 2 & 3). The three runs and their respective outcomes are summarized in Table 1. After treatment application, receiver corals were monitored for the onset of disease symptoms for ~4–6 weeks.

### DNA extractions and sequencing

Following the conclusion of Run 3, DNA was extracted from the frozen portion of all 0.22 µm pore size filters (from Runs 1, 2, and 3) using a DNEasy PowerBiofilm kit (Qiagen, Hilden, Germany). The manufacturer’s standard Quickstart Protocol (v. November 2016) was followed, except that bead beating was performed at 2,500 rpm on a BioSpec Products Mini Beadbeater instead of at 3,200 rpm on a PowerLyzer 24 Homogenizer. Blanks containing no filter were processed concurrent with sample extraction, and a positive control (MSA-3001 ABRF-MGRG 10 Strain Even Mix Genomic Material; ATCC, Manassas, Virginia) was incorporated for all downstream processing. Samples were shipped to the RTSF Genomics Core at Michigan State University for amplicon library preparation and sequencing.

Amplicon library preparation used the universal 16S (V4 region) primers 515F (5′-GTG CCA GCM GCC GCG GTA A-3′) and 806R (5′-GGA CTA CHV GGG TWT CTA AT-3′) (Caporaso et al., 2011) and followed Kozich et al.’s (2013) dual-index sequencing strategy. SequalPrep DNA Normalization plates (Invitrogen, Waltham, MA, USA) were used for batch normalization, with all resulting product then pooled and concentrated *via* QIAquick spin column (Qiagen, Hilden, Germany), then cleaned with magnetic SPRI beads (AMPure XP). Sequencing was performed on an Illumina MiSeq v2 Standard flow cell in a 2 × 250 bp paired end format with a v2 500 cycle reagent cartridge. Bases were called *via* Illumina Real Time Analysis (v1.18.54), with all output demultiplexed and converted to fastq files *via* Illumina Bcl2fastq (v2.20.0). All raw sequences are available through the NCBI Sequence Read Archive (PRJNA918331) and a U.S. Geological Survey data release (Kellogg, Evans & Voelschow, 2023).

### Bioinformatic processing and statistics

Demultiplexed sequences were imported into QIIME2 (v. 2022.2) (Bolyen et al., 2019) and denoised with DADA2 (Callahan et al., 2016) under default parameters, with truncation set to position 200. Taxonomy was assigned using a pre-trained naïve Bayes classifier SILVA-138-99-515-806 (Bokulich et al., 2018; Robeson et al., 2021), and an unrarefied amplicon sequence variant (ASV) table was generated; this table is publicly available through a USGS data release (Kellogg, Evans & Voelschow, 2023). Any sequences matching chloroplasts or mitochondria were removed from the dataset, and only ASVs assigned at the domain level (Bacteria or Archaea) were retained. Another unrarefied ASV table with these non-target ASVs removed was generated (also publicly available through a USGS data release (Kellogg, Evans & Voelschow, 2023)). A phylogenetic tree was generated using MAFFT (v. 7.0) (Katoh & Standley, 2013) and FastTree 2 (Price, Dehal & Arkin, 2010). Coral-derived samples with fewer than 300 raw sequences associated (*n* = 5) were considered to have failed sequencing and were removed from the dataset prior to alpha and beta-diversity analyses, as were quality controls (reagent controls and mock communities; *n* = 9).

Diversity analyses were conducted through the q2-diversity plugin. The “core-metrics-phylogenetic” method was used to rarefy the remaining samples (*n* = 51) to a depth of 19,051 sequences, and to generate Emperor principal coordinates analyses (PCoA) plots based on weighted and unweighted UniFrac distance matrices (Lozupone & Knight, 2005) in order to visualize the relationships between samples. The weighted UniFrac accounts for the greater influence of more abundant members of the microbial community in shaping community structure, while the unweighted UniFrac lends equal weight to all components, allowing for more influence by rarer constituents. Rarefaction curves were created to confirm this sampling depth was adequate in capturing the diversity associated with all samples.

For alpha diversity, metrics for richness (observed features), evenness (Pielou’s evenness; Pielou, 1966), and diversity (Shannon’s diversity index; Shannon, 1948) and Faith’s phylogenetic diversity (Faith, 1992) were calculated and statistically analyzed using Kruskal–Wallis tests and Benjamini–Hochberg false discovery rate (FDR) corrections (Benjamini & Hochberg, 1995). For beta diversity, permutational multivariate analyses of variance (PERMANOVA) tests were conducted based on the weighted UniFrac distance matrix, with pairwise PERMANOVA tests performed for all significant results and Benjamini–Hochberg FDR corrections applied to all pairwise comparison *p*-values. Permutational multivariate analyses of dispersion (PERMDISP) tests were performed to determine whether any significant PERMANOVA tests stemmed from unequal within-group dispersion, and Benjamini-Hochberg FDR corrections were applied. Our QIIME2 workflow is also publicly available through a USGS data release (Kellogg, Evans & Voelschow, 2023).

### SCTLD signal detection

To assess the presence of a SCTLD signal within our mesocosms and identify linkages to prior SCTLD studies, sequences corresponding to previously identified possible bioindicators of SCTLD (Becker et al., 2021) were compared against sequences from all samples in our unrarefied dataset (including healthy mesocosms, diseased mesocosms, FSW, mock communities, and reagent controls) using the NCBI BLASTn sequence alignment service. Any ASVs exhibiting 100% sequence identity match and 100% query cover (126/126 identities, 0 gaps) were considered matches with SCTLD bioindicators. ASVs matching to SCTLD bioindicators were then assessed for their presence in previously examined SCTLD-diseased coral tissue/mucus (Meyer et al., 2019; Rosales et al., 2020; Clark et al., 2021), disease-associated sediments (Studivan et al., 2022b), or disease-associated biofilms (Evans, Paul & Kellogg, 2022) to further evaluate the presence of a SCTLD signal. A 100% sequence identity match with 100% query coverage (252/252 or 253/253 identities, depending on sequence length, and 0 gaps, or 227/227 identities and 0 gaps, depending on trim/truncation parameters) was considered to represent the same ASV for that study.

### Identifying additional SCTLD-related ASVs

In addition to identifying the linkages between this and prior SCTLD studies as described above, we also identified ASVs specifically associated with SCTLD in this study. To identify ASVs enriched in diseased compared to apparently healthy mesocosms, a differential abundance analysis was performed using ANCOM (analysis of composition of microbiomes; Mandal et al., 2015) within the q2-composition plugin in QIIME2. Non-coral mesocosm samples were first filtered from the dataset, and ASVs exhibiting fewer than 10 total reads across all samples and ASVs present in only one mesocosm sample were trimmed from the dataset prior to ANCOM analysis. Additionally, based on the results of the transmission experiment, ASVs consistently associated with all 0.22 µm filters that resulted in tissue loss visually consistent with SCTLD were identified.

## Results

### Transmission test

During the disease transmission monitoring period following Run 1 (4 weeks), 2 out of the 60 receiver corals developed tissue loss signs visually consistent with SCTLD (Table 1). Both corals had received a 0.22 µm pore size filter treatment (a 0.8 µm pore size filter was not employed during this run), sourced in both cases from diseased and VcpA+ *Montastraea cavernosa* colonies (sample IDs Mcav-17 and Mcav-18). During the monitoring period for Run 2 (6 weeks), 0 out of the 100 receiver corals developed SCTLD (Table 1). However, direct contact challenges with diseased corals subsequently revealed that the receiver corals used in Run 2 were largely resistant to the disease; of the seven receiver corals (representing seven genotypes) challenged in direct contact with SCTLD-infected corals, one developed signs consistent with SCTLD infection (*i.e*., tissue loss) within 9 days, three became infected ~2 months later, and three never exhibited signs of infection. During the monitoring period following Run 3 (5 weeks), 20 out of the 84 receiver corals, spanning numerous treatment types (0.8, 0.22, and 0.025 µm pore size filters, and TFF filtrate) and including treatments sourced from both healthy and diseased donor corals, experienced some degree of tissue loss (Table 1). The three runs and their respective transmission outcomes, along with characteristics of the donor corals that contributed to a tissue loss response in receiver corals, are summarized in Table 1.

### Prokaryotic community characterization

In total, we obtained 9,181,396 raw sequences from 65 samples, with 8,534,232 of these sourced from coral mesocosms (*n* = 55), 621,755 from mock communities (*n* = 3), 21,631 from the seawater control (*n* = 1), and 3,778 from reagent blanks (*n* = 6). Five coral-derived samples (McH-104, Mcav-2SQ, CnH-101-Mar, CnD-22, and McH-103-4) returned fewer than 300 raw sequences per sample and were considered “failed” samples and removed from analysis. The remaining 50 coral-derived samples included 8,533,720 raw sequences. Of these, 7,493,038 remained following bioinformatic processing, which spanned 13,309 amplicon sequence variants (ASVs). A total of 1,196 sequences spanning 53 ASVs were found in reagent blanks (*n* = 5 blanks remained following denoising), with just 38 of these ASVs overlapping with coral mesocosm samples, indicating most mesocosm ASVs were not reagent contaminants.

**Figure 2:**
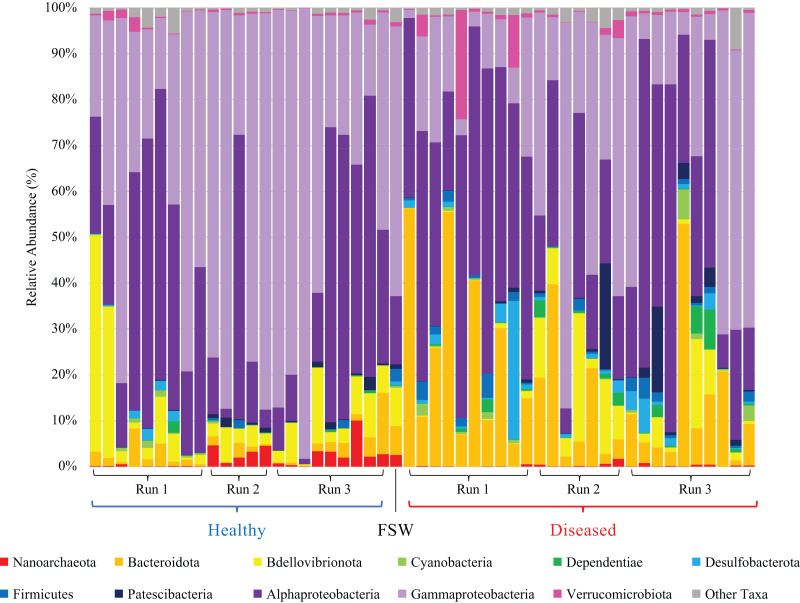
Relative abundance of prokaryotic taxa in healthy (blue bracket, left side) and diseased (red bracket, right side) coral mesocosms across all three experimental runs. Phylum-level taxonomic classifications are shown, except for Proteobacteria which was subdivided into major classes Alphaproteobacteria and Gammaproteobacteria, Magnetococcia, and “blank” (*i.e*., Proteobacteria unassigned at the class level). “Other Taxa” includes all taxa representing <0.5% average relative abundance across all coral samples, which included Magnetococcia and unassigned Proteobacteria. “FSW” represents the microbial community present in a comparable quantity (~18 L) of the filtered seawater used to fill the coral mesocosms. Taxa are stacked in reverse order as listed in the figure legend, as read from left to right, starting with “Nanoarchaeota” (red) at the bottom of the bar plots and ending with “Other Taxa” (gray) at the top of the plots.

**Figure 3:**
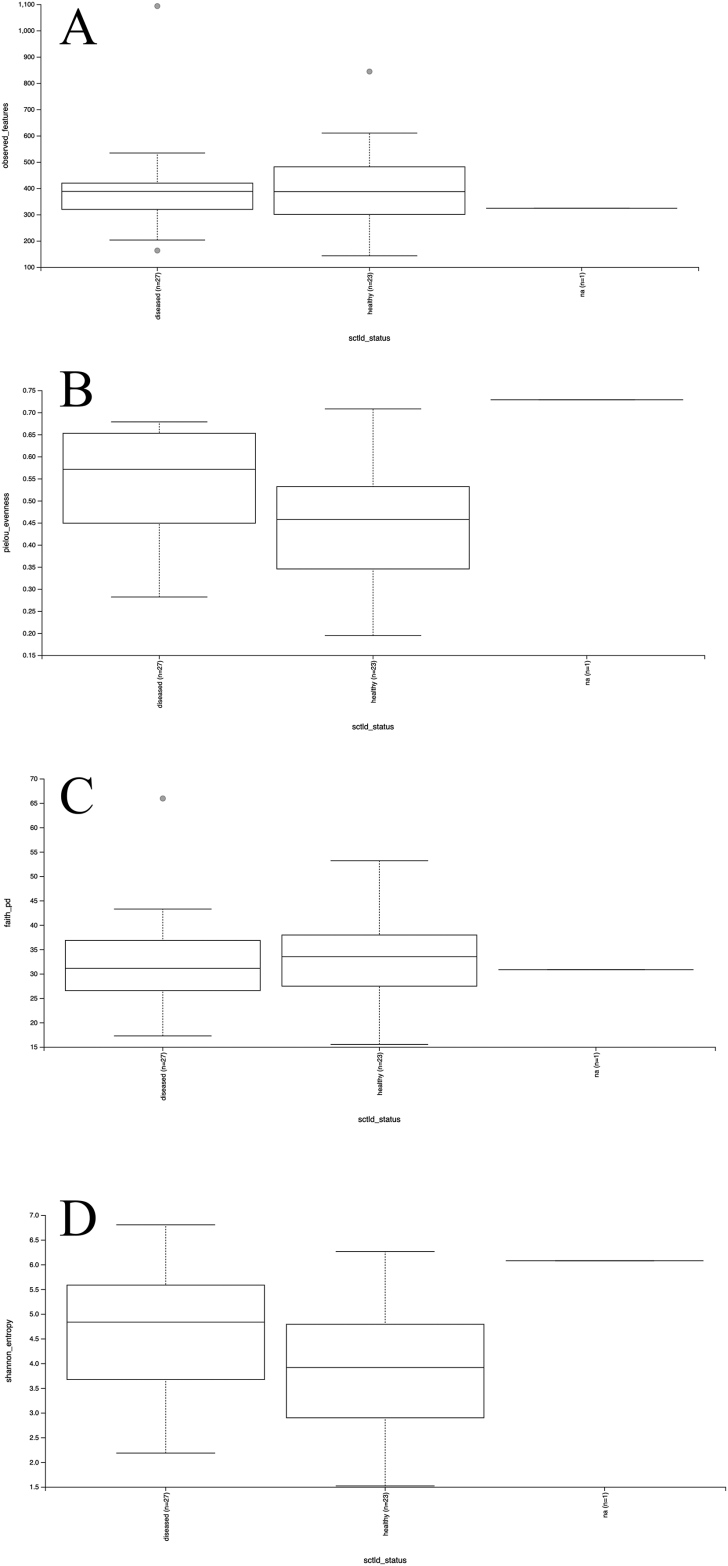
Boxplots for alpha diversity metrics. Plots for alpha diversity metrics richness (observed features; A), evenness (Pielou’s evenness; B), and Faith’s phylogenetic diversity (Faith’s PD; C) and Shannon’s diversity index (Shannon; D) are shown for diseased and healthy mesocosms (“diseased” and “healthy,” respectively), and filtered seawater (“na”).

Mesocosm prokaryotic communities exhibited high diversity, spanning 10 archaeal and 43 bacterial phyla (Fig. 2). The mesocosms were dominated by Proteobacteria, predominantly Alpha- and Gammaproteobacteria. Some general trends were apparent between diseased and healthy coral-shed prokaryotic communities. For example, diseased samples generally included greater relative abundances of Bacteroidota (~18% average relative abundance in diseased mesocosms compared to ~3% in healthy mesocosms), while healthy samples generally included greater relative abundances of Nanoarchaeota (~2% average relative abundance in healthy mesocosms compared to ~0.2% in diseased mesocosms). Diseased communities also generally appeared to deviate more from the “background” (filtered seawater) prokaryotic community. However, some effects of experimental run were also apparent (Fig. 2). Alpha diversity analyses indicated a significant difference (Kruskal–Wallis; *p* < 0.05) in evenness and diversity (Shannon’s diversity index) between diseased (*n* = 27) and healthy (*n* = 23) mesocosms (Fig. 3); however pairwise Kruskal–Wallis tests revealed no significant differences between mesocosm types following *p*-value correction (Table 2). No other variables examined, including species (conditions *C. natans* (CNAT), *Diploria labyrinthiformis* (DLAB), *M. cavernosa* (MCAV), *Orbicella faveolata* (OFAV), *P. strigosa* (PSTR), or seawater (FSW)), species + disease state (conditions diseased or healthy CNAT, DLAB, MCAV, OFAV, or PSTR, or FSW), experimental run (conditions Run 1, Run 2, or Run 3), or experimental run + disease state (conditions Runs 1, 2, or 3 diseased or healthy coral, and Run 3 FSW) exhibited significant differences in alpha diversity (Fig. 3; Table 2).

**Table 2:** Comparison of alpha diversity based on different characteristics of donor corals.

Metric	Comparison			Kruskal–Wallis		
				H	*p*-value	q-value
Richness (Observed Features)	Species			6.19	0.29	--
	Disease State			0.64	0.73	--
	Experimental Run			0.71	0.70	--
	Species + Disease State			9.28	0.51	--
	Experimental Run + Disease State			1.79	0.94	--
Evenness (Pielou’s)	Species			8.75	0.12	--
	Disease State			8.35	0.02*	--
	*Diseased*		*Healthy*	*5.69*	0.02*	*0.05*
	*Diseased*		*FSW*	*2.79*	*0.09*	*0.10*
	*Healthy*		*FSW*	*2.76*	*0.10*	*0.10*
	Experimental Run			0.16	0.92	--
	Species + Disease State			14.91	0.14	--
	Experimental Run + Disease State			10.67	0.10	--
Diversity (Faith’s PD)	Species			6.76	0.24	--
	Disease State			0.41	0.82	--
	Experimental Run			0.30	0.86	--
	Species + Disease State			11.24	0.34	--
	Experimental Run + Disease State			1.24	0.97	--
Diversity (Shannon)	Species			8.76	0.12	--
	Disease State			6.88	0.03*	--
	*Diseased*	*Healthy*		*4.63*	0.03*	*0.09*
	*Diseased*	*FSW*		*2.39*	*0.12*	*0.13*
	*Healthy*	*FSW*		*2.30*	*0.13*	*0.13*
	Experimental Run			0.36	0.83	--
	Species + Disease State			13.41	0.20	--
	Experimental Run + Disease State			9.79	0.13	--

**Note:**

Differences in metrics for richness, evenness, and diversity (Faith’s phylogenetic diversity (“Faith’s PD”) and Shannon’s diversity index (“Shannon”)) were assessed based on Species (conditions *Colpophyllia natans* (CNAT), *Diploria labyrinthiformis* (DLAB), *Montastraea cavernosa* (MCAV), *Orbicella faveolata* (OFAV), *Pseudodiploria strigosa* (PSTR), or filtered seawater (FSW)), Disease State (conditions diseased, healthy, or seawater), Experimental Run (conditions Run 1, Run 2, or Run 3), Species + Disease State (conditions diseased or healthy CNAT, DLAB, MCAV, OFAV, or PSTR, or FSW), and Experimental Run + Disease State (conditions Runs 1, 2, or 3 diseased or healthy coral, and Run 3 FSW). All significant results (*p* or q < 0.05) are indicated by asterisks (*). Pairwise Kruskal–Wallis tests, conducted for all significant results, are indicated in italics. “–” indicates value not calculated.

**Figure 4:**
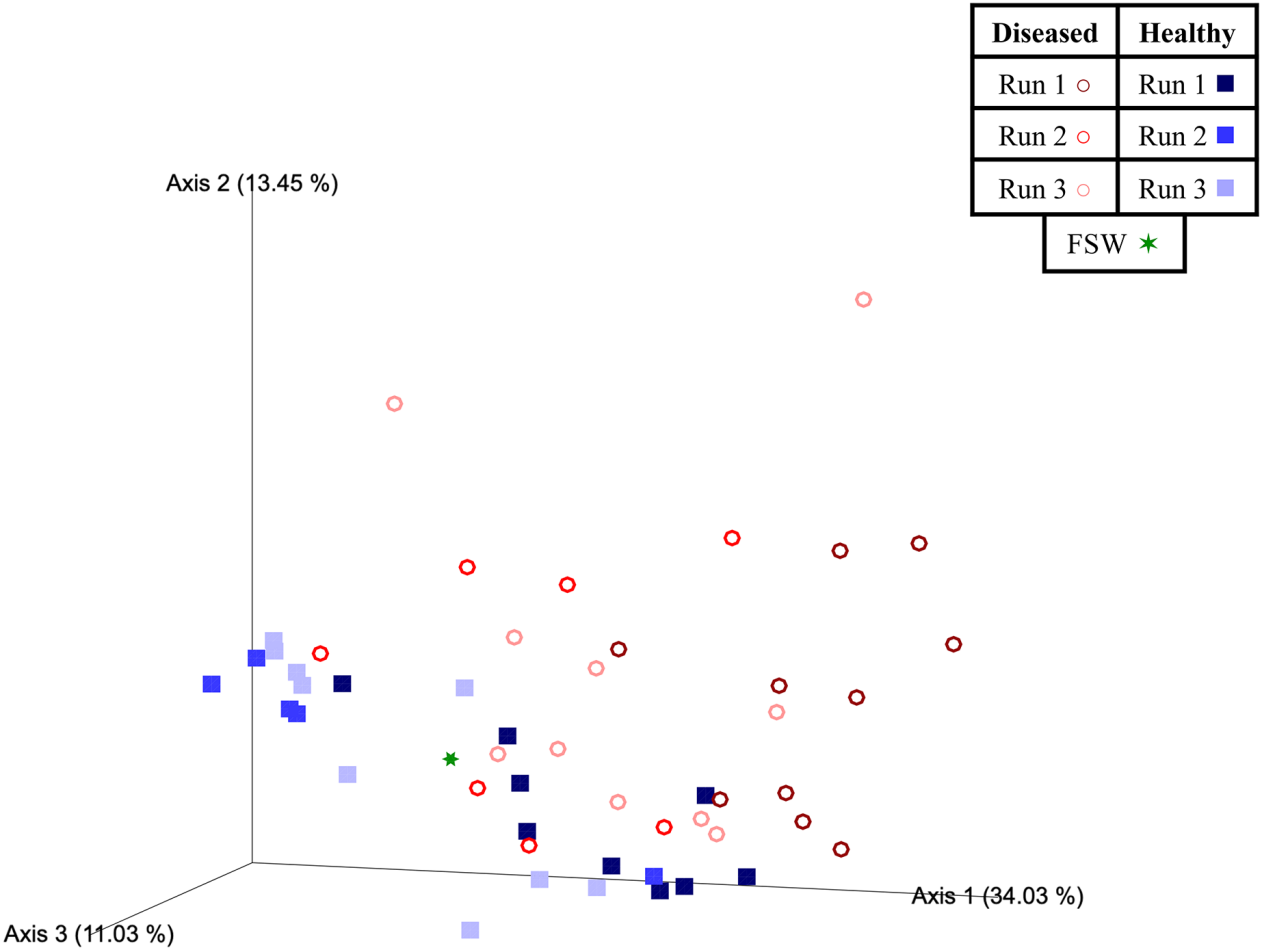
Two-dimensional Emperor principal coordinates analysis plots based on a weighted UniFrac distance matrix. Plots depict the relationships between the microbial communities sourced from different mesocosm types, including diseased mesocosms (circle shapes) from Runs 1 (maroon circles), 2 (red circles), and 3 (pink circles), healthy mesocosms (square shapes) from Runs 1 (navy blue squares), 2 (royal blue squares), and 3 (light blue squares), and filtered seawater (FSW) sampled during Run 3 (green star shape). Plots include axes for principal coordinates 1 and 2 (A) and 2 and 3 (B). Percentage values located on axes indicate the percent variation explained by that axis.

Beta diversity analyses indicated that significant differences (PERMANOVA; *p* < 0.05) in prokaryotic community structure existed between mesocosm samples based on multiple factors, including disease state, species, and experimental run (Table 3). For species (PERMANOVA; pseudo-F = 1.84; *p* = 0.013), pairwise tests revealed significant differences between OFAV and PSTR following *p*-value correction (q = 0.045; Table 3). A PERMDISP test further revealed significant differences in dispersion existed within species (PERMDISP; F-value = 5.69; *p* = 0.029; Table 3), with pairwise tests revealing a significant difference in dispersion between only CNAT and DLAB, following *p*-value correction (q = 0.030; Table 3). Significant differences in beta diversity were also detected between diseased (*n* = 27) and healthy (*n* = 23) coral mesocosms (PERMANOVA; pseudo-F = 4.80; *p* = 0.001; Table 3). The FSW microbial community could be expected to be present in all mesocosms, especially those from Run 3 (during which the FSW microbial community was sampled), however the small FSW sample size (*n* = 1) precluded our ability to make robust statistical comparisons. Nevertheless, the weighted UniFrac Emperor PCoA plots revealed that FSW clustered somewhat in the middle of healthy and diseased mesocosm samples (Fig. 4).

**Table 3:** Results from permutational multivariate analyses of variance (PERMANOVA) tests and permutational multivariate analyses of dispersion (PERMDISP) tests.

Comparison		PERMANOVA			PERMDISP		
		Pseudo-F	*p*-value	q-value	F-value	*p*-value	q-value
**Species**		1.84	0.013*	–	5.69	0.029*	–
CNAT	DLAB	2.90	0.018*	0.086	14.91	0.002*	0.030*
	MCAV	0.86	0.533	0.615	1.01	0.357	0.551
	OFAV	2.49	0.023*	0.086	7.38	0.013*	0.065
	PSTR	1.27	0.231	0.385	1.56	0.380	0.551
	FSW	0.83	0.677	0.725	18.84	0.227	0.435
DLAB	MCAV	2.47	0.066	0.198	5.94	0.013*	0.065
	OFAV	6.40	0.009*	0.068	2.37	0.102	0.306
	PSTR	2.21	0.113	0.242	2.66	0.090	0.306
	FSW	2.46	0.339	0.462	0.69	1.000	1.000
MCAV	OFAV	1.28	0.274	0.411	2.01	0.162	0.405
	PSTR	1.36	0.201	0.377	0.16	0.710	0.819
	FSW	0.86	0.414	0.518	9.78	0.842	0.902
OFAV	PSTR	3.69	0.003*	0.045*	0.70	0.404	0.551
	FSW	2.05	0.108	0.242	11.73	0.232	0.435
PSTR	FSW	0.68	0.828	0.828	8.13	0.671	0.819
**Disease State**		4.80	0.001*	–	9.09	0.235	–
Diseased	Healthy	8.60	0.001*	0.003*	–	–	–
Diseased	FSW	1.12	0.327	0.327	–	–	–
Healthy	FSW	1.20	0.237	0.327	–	–	–
**Experimental Run**		4.18	0.001*	–	2.71	0.065	–
Run 1	Run 2	6.20	0.001*	0.002*	–	–	–
Run 1	Run 3	5.73	0.001*	0.002*	–	–	–
Run 2	Run 3	1.05	0.347	0.347	–	–	–
**Experimental Run** **+ Disease State**		4.22	0.001*	–	3.54	0.145	–
Run 1, Diseased	Run 1, Healthy	7.02	0.001*	0.005*	–	–	–
	Run 2, Diseased	5.21	0.001*	0.005*	–	–	–
	Run 2, Healthy	13.62	0.006*	0.013*	–	–	–
	Run 3, Diseased	4.24	0.001*	0.005*	–	–	–
	Run 3, Healthy	11.62	0.002*	0.006*	–	–	–
	Run 3, FSW	3.25	0.100	0.150	–	–	–
Run 1, Healthy	Run 2, Diseased	3.19	0.005*	0.012*	–	–	–
	Run 2, Healthy	5.43	0.012*	0.023*	–	–	–
	Run 3, Diseased	4.74	0.001*	0.005*	–	–	–
	Run 3, Healthy	4.64	0.003*	0.008*	–	–	–
	Run 3, FSW	2.36	0.108	0.151	–	–	–
Run 2, Diseased	Run 2, Healthy	3.20	0.024*	0.042*	–	–	–
	Run 3, Diseased	1.38	0.184	0.242	–	–	–
	Run 3, Healthy	2.13	0.066	0.107	–	–	–
	Run 3, FSW	0.85	0.479	0.479	–	–	–
Run 2, Healthy	Run 3, Diseased	5.04	0.002*	0.006*	–	–	–
	Run 3, Healthy	1.14	0.305	0.377	–	–	–
	Run 3, FSW	2.31	0.354	0.413	–	–	–
Run 3, Diseased	Run 3, Healthy	4.53	0.002*	0.006*	–	–	–
	Run 3, FSW	0.97	0.470	0.479	–	–	–
Run 3, Healthy	Run 3, FSW	1.02	0.389	0.430	–	–	–

**Note:**

Tests are based on a weighted UniFrac distance matrix, with pairwise PERMANOVA tests conducted for all significant results. Comparisons were performed based on Species (conditions *Colpophyllia natans* (CNAT), *Diploria labyrinthiformis* (DLAB), *Montastraea cavernosa* (MCAV), *Orbicella faveolata* (OFAV), *Pseudodiploria strigosa* (PSTR), or filtered seawater (FSW)), Disease State (conditions diseased, healthy, or seawater), Experimental Run (conditions Run 1, Run 2, or Run 3), Species + Disease State (conditions diseased or healthy CNAT, DLAB, MCAV, OFAV, or PSTR, or FSW), and Experimental Run + Disease State (conditions Runs 1, 2, or 3 diseased or healthy coral, and Run 3 FSW). Asterisks (*) indicate significant results (*p* < 0.05, q < 0.05). “–” indicates value not calculated.

**Figure 5:**
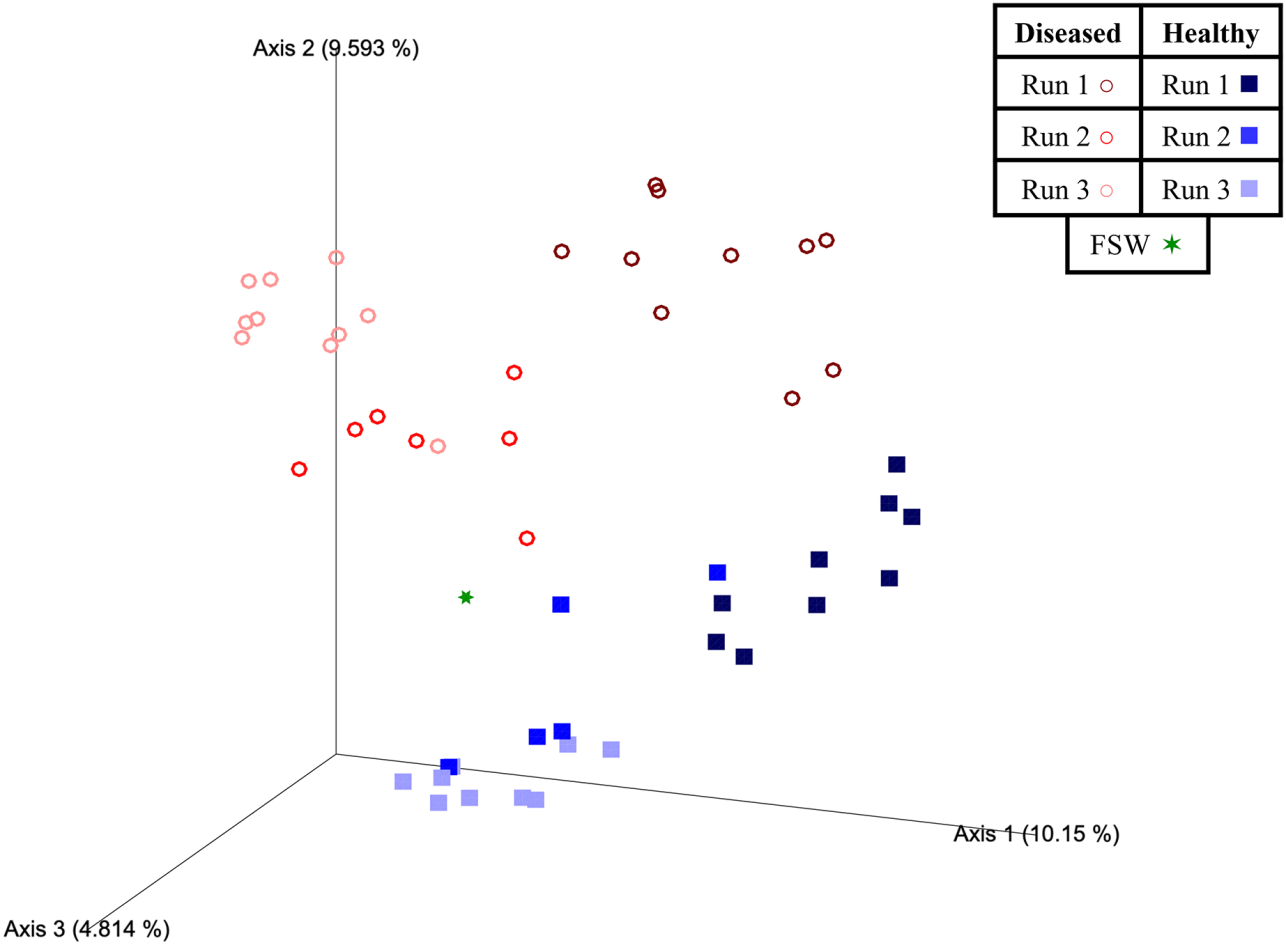
Emperor principal coordinates analysis plots based on an unweighted UniFrac distance matrix. Plots depict the relationships between the microbial communities sourced from different mesocosm types, including diseased mesocosms (circle shapes) from Runs 1 (maroon circles), 2 (red circles), and 3 (pink circles), healthy mesocosms (square shapes) from Runs 1 (navy blue squares), 2 (royal blue squares), and 3 (light blue squares), and filtered seawater (FSW) sampled during Run 3 (green star shape). Plots include axes for principal coordinates 1 and 2 (A) and 2 and 3 (B). Percentage values located on axes indicate the percent variation explained by that axis.

Experimental run was also found to significantly influence beta diversity (PERMANOVA; pseudo-F = 4.18; *p* = 0.001), with pairwise comparisons revealing that Runs 2 and 3 (November 2020 and March 2021, respectively) were significantly different from Run 1 (October 2019; q = 0.002 for both comparisons), but not from each other (q = 0.347; Table 3). When we further subdivided experimental run into healthy and diseased components (conditions Run 1, diseased; Run 1, healthy; Run 2, diseased; Run 2, healthy; Run 3, diseased; and Run 3, healthy), we again found an overall significant influence on prokaryotic community structure (PERMANOVA; pseudo-F = 4.22; *p* = 0.001). Pairwise comparisons revealed that for each experimental run (1, 2, and 3), the diseased mesocosms were significantly different from the corresponding healthy mesocosms for that run (q < 0.05 for all comparisons; Table 3). In addition, healthy and diseased Run 1 coral mesocosms were significantly different from all other runs + disease state combinations (q < 0.05 for all comparisons; Table 3). Run 2, diseased mesocosms were not significantly different from either Run 3, diseased or Run 3, healthy mesocosms (q = 0.24 and 0.11, respectively). Run 2, healthy mesocosms were similarly not significantly different from Run 3, healthy mesocosms (q = 0.38) but were significantly different from Run 3, diseased mesocosms (q = 0.006; Table 3). The unweighted UniFrac Emperor PCoA plots revealed that, when rare microbiome constituents are given equal weight to highly abundant members, samples seem to cluster much more tightly by experimental run + disease state (Fig. 5). Besides species, no significant differences in dispersion were detected for any of the other comparisons (PERMDISP, *p* > 0.05; Table 3).

To determine the strength of a SCTLD signal within our disease-associated mesocosms, we searched for 25 previously identified SCTLD “bioindicator” ASVs (Becker et al., 2021) across all mesocsm types and determined that 22 of these bioindicators were present within our dataset (including diseased and healthy mesocosms, in the filtered seawater, and in control samples (*i.e*., reagent controls and mock communities); Table 4). These 22 bioindicator ASVs corresponded to 38 mesocosm ASVs (due to differences in sequence length) that were 100% matches for the overlapping sequence region. Of these, 20 were found exclusively in diseased mesocosms (*i.e*., not detected in healthy mesocosms, FSW, or controls), two were exclusively detected in healthy mesocosms, two were exclusively found in FSW, and 0 were exclusive to the controls (Table 4). In fact, only one of these ASVs was detected in control samples—ASV29 had six reads in a single mock community sample (Table 4). Of the 14 ASVs detected in more than one sample type (healthy or diseased mesocosms, FSW, or controls), three were in greater abundance in healthy mesocosms (*n* = 23) than diseased mesocosms (*n* = 27; Table 4). Sequences for the 22 bioindicator ASVs from Becker et al. (2021) and the 38 corresponding ASVs from this study are provided in the USGS data release (Kellogg, Evans & Voelschow, 2023).

**Table 4:** Amplicon sequence variants (ASVs) detected in this study that represent 100% sequence identity matches for the overlapping region of stony coral tissue loss disease (SCTLD) bioindicator ASVs (Becker et al., 2021).

ASV ID		Abundance				Taxonomic ID	Previously detected in SCTLD-associated samples?				
SCTLD Bioindicator (Becker et al., 2021)	This study	Diseased mesocosms (*n* = 27)	Healthy mesocosms (*n* = 23)	FSW (*n* = 1)	Controls (*n* = 8)	Lowest classification	Clark et al. (2021)	Evans et al. (2022)	Meyer et al. (2019)	Rosales et al. (2020)	Studivan et al. (2022b)
ASV13	asv29	41,905	444	41	6	g. *Halodesulfovibrio*	Y	Y	Y	Y	Y
	asv1368	135	–	–	–		–	–	–	–	–
ASV20	asv169	2,081	2,445	–	–	g. *Vibrio*	Y	–	Y	Y	Y
	asv5469	–	13	–	–		–	–	–	–	–
ASV21	asv1396	129	–	–	–	g. *Malaciobacter*	Y	Y	Y	Y	–
ASV25	asv267	1,103	1,206	–	–	g. *Vibrio*	Y	Y	Y	Y	Y
	asv13380	–	–	16	–		–	–	–	–	–
ASV26	asv63	17,615	–	–	–	g. *Roseimarinus*	Y	Y	Y	Y	–
ASV34	asv546	683	–	–	–	f. Rhodobacteraceae	Y	Y	Y	Y	–
ASV36	asv614	555	4	–	–	s. *Tepidibacter mesophilus*	Y	Y	Y	Y	Y
ASV39	asv104	8,643	8	–	–	g. *Marinifilum*	Y	Y	Y	–	–
	asv236	3,032	–	–	–		Y	–	Y	–	–
	asv762	384	–	–	–		–	–	–	–	–
	asv2072	64	–	–	–		–	–	–	–	–
	asv6941	8	–	–	–		–	–	–	–	–
ASV44	asv554	660	–	–	–	g. *Fusibacter*	Y	–	Y	Y	Y
	asv9528	5	–	–	–		–	–	–	–	–
ASV48	asv594	591	–	–	–	g. *Halarcobacter*	Y	–	Y	Y	Y
ASV52	asv852	117	207	–	–	g. *Algicola*	Y	Y	Y	–	Y
	asv4963	15	–	–	–		–	–	–	–	–
	asv7108	5	3	–	–		Y	Y	Y	Y	Y
	asv13412	–	–	9	–		–	–	–	–	–
ASV54	asv678	472	–	–	–	g. *Vibrio*	Y	Y	Y	Y	Y
ASV60	asv47	13,626	8,458	40	–	g. *Shimia*	Y	Y	Y	Y	Y
	asv1090	210	–	–	–		–	–	–	–	–
ASV67	asv449	620	348	–	–	g. *Vibrio*	Y	–	Y	Y	Y
ASV96	asv2749	–	40	–	–		Y	–	Y	Y	–
ASV101	asv893	246	55	–	–	f. Arcobacteraceae	Y	Y	Y	Y	Y
ASV111	asv189	4,026	31	–	–	f. Rhodobacteraceae	Y	Y	Y	Y	–
ASV135	asv282	2,049	19	–	–	g. *Fusibacter*/*Clostridiales*	Y	Y	Y	Y	Y
	asv6247	10	–	–	–		–	–	–	–	
	asv11943	3	–	–	–		–	–	–	–	–
ASV185	asv12018	3	–	–	–	s. *Desulfovibrio salexigens*	Y	Y	Y	–	–
ASV226	asv203	3,630	–	–	–	g. *Cohaesibacter*	Y	Y	Y	Y	Y
ASV263	asv730	413	–	–	–	f. Arcobacteraceae	–	–	Y	Y	Y
ASV275	asv71	15,542	78	–	–	g. *Fusibacter*/ *Acidaminobacter* sp.	Y	Y	–	–	–
	asv626	513	23	–	–		Y	Y	Y	Y	Y
	asv2923	36	–	–	–		Y	–	–	–	–
**Total Reads (all ASVs)**		3,782,518	3,710,520	19,051	455,834						

**Note:**

The lowest taxonomic classification (f = family, g = genus, s = species) of each ASV is indicated, along with the total number of reads for each ASV within each mesocosm type (diseased or healthy), filtered seawater (FSW), or controls (reagent controls and mock communities), where “*n*” indicates the number of samples corresponding to that sample type and “–” indicates the ASV was not detected in that sample type. Whether the ASVs have been previously observed in SCTLD lesion tissue/mucus (Meyer et al., 2019; Rosales et al., 2020; Clark et al., 2021), SCTLD-associated biofilms (Evans, Paul & Kellogg, 2022), or SCTLD-associated sediments (Studivan et al., 2022b) is also indicated, with “Y” indicating that the ASV was previously detected in at least one SCTLD-associated sample from that study, and “–” indicating that the ASV was not detected in any SCTLD-associated samples from that study. “Total Reads (all ASVs)” indicates the total number of reads across all ASVs (*i.e*., not just bioindicator ASVs) associated with that sample type.

To further assess the strength of a SCTLD signal within our dataset, all 38 bioindicator-matching ASVs were then compared against datasets from studies investigating bacterial communities associated with SCTLD-infected coral tissue/mucus (Meyer et al., 2019; Rosales et al., 2020; Clark et al., 2021), SCTLD-associated sediments (Studivan et al., 2022b), and SCTLD-associated biofilms (Evans, Paul & Kellogg, 2022). Numerous 100% identical sequences were identified associated with disease samples across these different studies (Table 4).

We also identified any ASVs that were consistently detected across all five 0.22 µm pore size filters that resulted in tissue loss visually consistent with SCTLD in healthy receiver corals. Fifteen ASVs matched this criterion (Table 5). Two of these (ASV29 and ASV47) were also matches for SCTLD bioindicators (Table 4), and three (ASV42, ASV142, and ASV250) were found exclusively in diseased mesocosms and not healthy mesocosms, filtered seawater, or any of the control samples (reagent blanks or mock communities; Table 5). The differential abundance analysis further identified seven ASVs that were enriched in SCTLD-associated mesocosms compared to apparently healthy mesocosms (Table 6). Of these, three (ASV29, ASV71, ASV203) were also matches for SCTLD bioindicators (Table 4). The full ANCOM results are publicly available through a USGS data release (Kellogg, Evans & Voelschow, 2023).

**Table 5:** Amplicon sequence variants (ASVs) found in all five of the 0.22 µm pore size filters that elicited signs consistent with SCTLD in healthy receiver corals.

ASV ID	Taxonomic ID		Total # of reads									
	Phylum	Lowest ID	Mcav-17	Mcav-18	CnD-20	CnD-23	PsD-5	Transmission samples	All diseased mesocosms	All healthy mesocosms	FSW	Controls
ASV1	Proteobacteria	f. Alteromonadaceae	100	945	3,567	18	14,784	19,414	230,420	753,712	967	521
ASV5	Bacteroidota	s. *Phaeodactylibacter xiamenensis*	5,524	29,230	29	19	42	34,844	218,627	943	27	21
ASV12	Proteobacteria	g. *Cognatishimia*	6,136	163	1,339	8,922	110	16,670	67,134	41,437	74	0
ASV16	Proteobacteria	g. *Litoricola*	3,387	1,892	152	12	9,232	14,675	74,746	9,873	223	4
ASV29	Desulfobacterota	g. *Halodesulfovibrio*	328	1,561	1,063	2,577	145	5,674	41,905	444	41	6
ASV42	Bdellovibrionota	g. *Pseudobacteriovorax*	20	72	34	343	11	480	23,907	0	0	0
ASV44	Proteobacteria	f. Rhodobacteraceae	230	866	118	163	103	1,480	10,816	12,202	0	0
ASV47	Proteobacteria	g. *Shimia*	24	65	300	1,645	127	2,161	13,626	8,458	40	0
ASV100	Proteobacteria	f. Rhodobacteraceae	33	105	1,030	75	45	1,288	8,682	220	0	0
ASV142	Desulfobacterota	g. *Halodesulfovibrio*	176	312	791	23	197	1,499	5,464	0	0	0
ASV198	Proteobacteria	f. Rhodobacteraceae	200	55	80	952	103	1,390	2,947	777	64	0
ASV229	Proteobacteria	f. Rickettsiaceae	206	13	535	2	16	772	3,074	16	0	0
ASV250	Proteobacteria	f. Rhodobacteraceae	25	265	44	42	33	409	2,638	0	0	0
ASV294	Firmicutes	g. *Vallitalea*	143	435	12	10	14	614	1,944	23	0	0
ASV311	Proteobacteria	o. Rhodospirillales	28	12	138	115	146	439	1,790	2	9	0

**Note:**

The taxonomic identity corresponding to each of these ASVs is indicated at the phylum level, along with the lowest achieved classification (o = order, f = family, g = genus, s = species). The total # of reads for each ASV are given for: the five transmission filters (sourced from donor corals Mcav-17, Mcav-18, CnD-20, CnD-23, and PsD-5); the sum total for all five transmission samples; the sum total for all diseased mesocosms (*n* = 27); the sum total for all healthy mesocosms (*n* = 23); the total in the filtered seawater sample (FSW; *n* = 1); and the sum total in all controls (mock communities (*n* = 3) and reagent blanks (*n* = 5); combined *n* = 8).

**Table 6:** Amplicon sequence variants (ASVs) identified by differential abundance analysis (ANCOM) as enriched in SCTLD-associated or apparently healthy mesocosms.

ASV ID	ANCOM	Taxonomic ID		Total # of reads			
	W	Phylum	Lowest ID	All diseased mesocosms	All healthy mesocosms	FSW	Controls
ASV8	3312	Bdellovibrionota	g. *Pseudobacteriovorax*	300	163,646	54	0
ASV29	3274	Desulfobacterota	g. *Halodesulfovibrio*	41,905	444	41	6
ASV34	3152	Nanoarchaeota	f./g. *SCGC_AAA286-E23*	2,476	32,961	16	0
ASV42	3216	Bdellovibrionota	g. *Pseudobacteriovorax*	23,907	0	0	0
ASV52	3259	Proteobacteria	f. Hyphomonadaceae	19,316	459	0	0
ASV71	3012	Firmicutes	g. *Fusibacter*	15,542	78	0	0
ASV75	3233	Proteobacteria	g. *Porticoccus*	14,841	0	0	0
ASV88	3133	Bdellovibrionota	g. *Peredibacter*	1,351	9,527	0	0
ASV142	2985	Desulfobacterota	g. *Halodesulfovibrio*	5,464	0	0	0
ASV187	3037	Proteobacteria	f. Halieaceae	18	4,058	0	0
ASV203	3184	Proteobacteria	g. *Cohaesibacter*	3,630	0	0	0
**Total Reads (all ASVs)**				3,782,518	3,710,520	19,051	455,834

**Note:**

“ASV ID” indicates the assigned ASV identity from this study. “W” is the ANCOM W statistic. The taxonomic identity corresponding to each of these ASVs is indicated at the phylum level, along with the lowest achieved classification (f = family, g = genus). The total # of reads for each ASV are given for: the sum total for all diseased mesocosms (*n* = 27); the sum total for all healthy mesocosms (*n* = 23); the total in the filtered seawater sample (FSW; *n* = 1); and the sum total in all controls (mock communities (*n* = 3) and reagent blanks (*n* = 5); combined *n* = 8). The total number of reads for the entire dataset (*i.e*., all ASVs) are also indicated for each sample type.

## Discussion

Here we employed a previously developed coral disease investigative methodology designed to reduce the background noise typically associated with coral tissue samples while isolating disease-associated microbes into different microbial size classes (Evans et al., 2022). We then combined this approach with experimental disease transmission tests in an attempt to identify which microbial size class, and by association which microbial group (*e.g*., microeukaryotes, bacteria, viruses, *etc*.) elicits SCTLD signs in healthy corals. The results of these transmission experiments were inconclusive. While the experimental outcome of Run 1 suggested microbes retained on the 0.22 µm pore size filters sourced from SCTLD-infected donor corals elicit a disease response in healthy receiver corals, this was the largest pore size filter employed following 200 µm pore size prefiltration during this experimental run, and therefore represented a mixed microbial community, potentially including microeukaryotes, bacteria, and large viruses. Consequently, additional runs were undertaken to further fractionate the mesocosm microbial communities into larger (0.8 µm pore size) and smaller (0.22 µm pore size) components and clarify the size class responsible for SCTLD transmission.

Subsequent runs employed receiver corals that were later determined to be either largely resistant to SCTLD (Run 2) or in a compromised state (Run 3), which complicated the results. Indeed, when representative receiver *O. faveolata* coral plugs from Run 2 (*n* = 7) were placed in direct contact with SCTLD-infected corals and monitored, only one became infected within a 10-day period. It took more than 8 weeks for three additional receiver corals to become infected, and three never showed any disease signs, suggesting Run 2 receivers were largely not susceptible to SCTLD. This is possibly because these corals were raised *in situ* in the SCTLD-endemic zone, suggesting these corals may have possessed an innate resistance to the disease. Run 1 receiver corals were also acquired from the same source, which may explain why we did not observe higher rates of transmission in this initial run.

Run 3 receiver corals were sourced from a land-based nursery stock (*i.e*., *ex situ*), but some corals in the tanks housing these corals experienced a tissue loss outbreak shortly prior to the start of our experiment. Although we used only apparently healthy individuals with no tissue loss signs in our study, these corals may have been asymptomatic for disease, with symptoms occurring in response to stress and created the mixed results across treatment types that we observed. Indeed, in some cases we even observed tissue loss in receiver corals that were exposed to filters sourced from healthy donors. Thus, although some microbes retained on the 0.22 µm pore size filters again appeared to transmit SCTLD in Run 3, so too did microbes retained on the 0.8 and 0.025 µm pore size filters, and the TFF filtrate.

Another caveat to consider is that, in addition to representing a mixed community of numerous different microbial size classes as described above, the microbes retained on the 0.22 µm pore size filters that transmitted disease in Run 1 were sourced from *M. cavernosa* lesions that tested positive for VcpA. While a previous study suggests that the bacterium that produces VcpA, *Vibrio coralliilyticus*, is not a primary pathogen of SCTLD in *M. cavernosa*, it is still capable of initiating tissue loss lesions at a low rate and appears to exacerbate SCLTD (Ushijima et al., 2020). This could suggest that this opportunistic pathogen, and other potential opportunists, may be needed for the visual signs of SCTLD to occur.

We also acknowledge that healthy and diseased donor corals differed in their source, which could account for some of the differences observed between healthy and diseased mesocosm prokaryotic communities. While all the healthy donor corals used in this experiment had been collected off reefs or from coral nurseries, then collectively held in the SMS healthy coral stock water tables for at least 8 months prior to the start of the experiment, diseased corals were collected off the reef and used in the experiment immediately. This option was chosen as an alternative to collecting apparently healthy individuals off the reef as some studies have suggested apparently healthy corals on the reef may still represent asymptomatic SCTLD infections (Work et al., 2021), and given their use as healthy controls in this experiment, we needed to ensure that healthy donor corals were truly healthy. However, while some research has suggested that the coral microbiome may not be substantially impacted by translocation from reef to aquaria (Damjanovic et al., 2020), other studies have found the opposite (Pratte, Richardson & Mills, 2015; Röthig et al., 2017), suggesting this *ex situ* holding period for our healthy corals could potentially have impacted the microbes associated with healthy compared to diseased donors. Further, for nursery-sourced corals, we do not know the original provenance of many of our healthy donors, as they were frequently opportunistically collected “rescue” colonies that may have been sourced from locations such as nearshore seawalls, docks, *etc*. Geographic location has also been shown to impact coral microbiomes (Littman et al., 2009) and may have further contributed to differences between healthy and diseased corals. Thus, it may be impossible to fully determine the individual influence of coral health status *vs*. collection source on prokaryotic community structure here. Nevertheless, the healthy mesocosms were not intended to represent true uninfected baseline coral microbiomes here; rather, they were included as controls to ensure that filters containing concentrated coral-associated microbes would not trigger a stress response unrelated to the disease, and to help identify ASVs that are common coral associates. Thus, in this context, potential alteration of the healthy corals’ microbiomes is not relevant to our broader findings.

Following the conclusion of the three transmission experiments, we chose to characterize the prokaryotic community retained on the 0.22 µm pore size filters, as this filter size exhibited apparent transmission in Run 1 and was the only treatment type employed in Run 3 in which healthy controls did not also elicit a disease response in receiver corals. Specifically we focused our investigation on the 16S rRNA gene (*i.e*., the prokaryotic component) because the vast majority of SCTLD-centric microbiological studies have described the associated bacterial/archaeal community (Meyer et al., 2019; Rosales et al., 2020; Becker et al., 2021; Clark et al., 2021; Iwanowicz et al., 2020; Evans, Paul & Kellogg, 2022; Evans et al., 2022; Huntley et al., 2022; Rosales et al., 2022; Rosales et al., 2023; Studivan et al., 2022b), which allowed us to extensively compare across studies that span different collection years, locations, and coral species.

At the time of our final experimental run (Run 3; March 2021), the TEM evidence inferring the possibility of an RNA virus linked to SCTLD had not yet been published (Work et al., 2021), and thus we did not preserve our samples for RNA analysis. However, we do note that the 0.22 and 0.8 µm pore size filters would have retained the virus-like particles (VLPs) of the size range (558–6,697 nm in length) observed by Work et al. (2021). Work et al. (2021) also noted that these VLPs were observed within coral endosymbionts (Symbiodiniaceae). Corals often will expel their Symbiodiniaceae (zooxanthellae) in times of stress (Jones, 1997). If the VLPs contained within the zooxanthellae are indeed the causative agent of SCTLD, could infected expelled zooxanthellae represent a possible vector for the disease? Zooxanthellae would be expected to be captured on the 0.8 µm pore size filters following our experimental design, except in Run 1 when a 0.8 µm pore size filter was not employed, and zooxanthellae would have been retained on the 0.22 µm pore size filters instead. The microbes retained on the 0.22 µm pore size filters in Run 1 did appear to transmit SCTLD to the receiver corals, and we observed apparent transmission among the microbes retained on the 0.8 µm pore size filters employed in Run 3 (along with other treatment types however, as noted above). Even if the infected zooxanthellae had lysed during sample processing, releasing their VLPs, these particles would still have been captured on the 0.8 or 0.22 µm pore size filters, as noted above. Thus, while the results of our transmission experiment are imperfect, we note that they could potentially support this hypothesis of a viral cause of SCTLD.

Becker et al. (2021) identified 25 bacterial ASVs as possible SCTLD “bioindicators” that were enriched in SCTLD lesions compared to healthy tissue, and many of which were also detected in seawater near the corals. To determine whether our coral mesocosms acquired a SCTLD “signal” when associated with diseased corals, we assessed whether these ASVs were present within our dataset. Given the difference in sequence length between the shorter bioindicator ASVs and our ASVs, we identified all ASVs that represented 100% identical sequence matches to bioindicators. Of the 25 SCTLD bioindicators, we detected 22 within our mesocosms. Bioindicators were represented by 38 ASVs, due to the difference in sequence length between bioindicators (126 bp) and this study (252–253 bp). We then further assessed the strength of a SCTLD signal by searching for all 38 ASVs corresponding to bioindicators within other SCTLD prokaryotic datasets (Meyer et al., 2019; Rosales et al., 2020; Clark et al., 2021; Evans, Paul & Kellogg, 2022; Studivan et al., 2022b), and identified all matches. Interestingly, where a bioindicator corresponded to multiple ASVs in this study, often just one of these bioindicator matches appeared in other studies, further underscoring the disease-specificity of many of these bioindicator ASVs. For example, bioindicator ASV13 (*Halodesulfovibrio*) matched to two ASVs from this study: ASV29 and ASV1368. However, while ASV29 was detected in disease-associated samples from all five comparison studies, ASV1368 was not found in diseased samples in any of them.

Whether any of these bioindicators are involved in disease causation or progression remains unclear. Some of the ASVs matching to bioindicators were found exclusively in diseased samples, or with greater numbers of reads compared to healthy mesocosms. For example, ASV63 (genus *Roseimarinus*), a match to bioindicator ASV26, included a total of 17,615 reads across the 27 diseased mesocosms, and was detected in none of the 23 healthy mesocosms. Such increases in diseased samples compared to healthy mesocosms could indicate that these ASVs are involved in disease causation or progression but could also simply mean that these ASVs are saprophytic opportunists, responding to the increased nutrients associated with the sloughing tissue associated with SCTLD progression. Other bioindicator ASVs, such as ASV20, ASV25, and ASV52, matched to ASVs that were in similar or greater abundance in healthy compared to diseased mesocosms, suggesting they are likely general coral associates, rather than specific to SCTLD. This can include genera that are commonly associated with coral diseases, such as *Vibrio* (bioindicators ASV20 and 25). Nevertheless, the considerable overlap overall between ASVs detected in this study and ASVs identified by prior SCTLD studies spanning different years, coral species, source material (coral tissue/mucus, sediments, or biofilms), and geographic regions (Meyer et al., 2019; Rosales et al., 2020; Becker et al., 2021; Clark et al., 2021; Evans, Paul & Kellogg, 2022; Studivan et al., 2022b) suggests that these ASVs may warrant additional consideration for their potential role in SCTLD causation and/or progression. Differential abundance analysis further identified seven ASVs from this study that were enriched in diseased compared to healthy meosocosms, with three of these also representing matches to SCTLD bioindicator ASVs: ASV29 (genus *Halodesulfovibrio*), ASV71 (genus *Fusibacter*), and ASV203 (genus *Cohaesibacter*).

Over the course of three experimental runs, five receiver corals treated with 0.22 µm pore size filters sourced from diseased donor corals developed tissue loss visually consistent with SCTLD. Though all five cases of putative disease transmission had other potentially confounding influences as discussed, we investigated whether any bacterial ASVs were consistently present on all five “transmission” filters, as these could potentially represent ASVs relevant to SCTLD causation or progression. Fifteen ASVs matched this criterion, though several were also of equal or greater abundance in healthy coral mesocosms (*e.g*., ASV44), suggesting they are more likely general coral symbionts rather than associated specifically with the disease. Some were also highly abundant within the FSW sample (*e.g*., ASVs 1, 12, 16), suggesting they may represent baseline bacteria that were present within the initial mesocosm environment. Some potential FSW baseline ASVs, such as ASV16, appeared in greater total abundance in diseased mesocosms compared to healthy mesocosms, suggesting these opportunists may have bloomed in the nutrient-rich environments created by sloughing diseased tissue. Most of the fifteen ASVs had few or no reads in the controls (mock communities + reagent blanks; *n* = 8 combined (one reagent blank was removed during rarefaction)) with the exception of ASV1; however, it is unclear, given the huge abundance of this ASV across all sample types, to what extent reads of this ASV within the controls may represent background noise from sequencing “bleed” (Nearing, Comeau & Langille, 2021). Several ASVs were also identified that were both present in all five transmission filters and unique to diseased mesocosms. ASV42 (genus *Pseudobacteriovorax*), ASV142 (genus *Halodesulfovibrio*), and ASV250 (family Rhodobacteraceae) were not detected in any of the healthy mesocosms, the FSW sample, or the controls, but were found in association with all five “transmission” 0.22 µm pore size filters. However, the relationship, if any, between these ASVs and SCTLD remains unclear. For example, while ASV42 may have been unique to disease mesocosms and present in all transmission-associated filters, other *Pseudobacteriovorax* ASVs (*e.g*., ASV8, this study) were detected almost exclusively in healthy and not diseased mesocosms (*n* = 163,646 and 300 total reads, respectively).

Rosales et al. (2023) performed a meta-analysis of existing SCTLD-associated 16S rRNA gene datasets and identified the order Peptostreptococcales-Tissierellales as potentially relevant to the disease (*i.e*., enriched in SCTLD lesions). In our study, 91 ASVs matched to this order, with nine of these also representing matches to bioindicator ASVs for the overlapping region (Becker et al., 2021). These 91 ASVs included 29,760 total reads across all diseased mesocosms, and just 480 reads across healthy mesocosms, and 12 reads in the FSW sample. Just 1 Peptostreptococcales-Tissierellales ASV was identified within a reagent blank sample, with just 13 reads detected, and this particular ASV was not found in any of the coral mesocosms or the FSW. However, as with the bioindicators, whether any of these ASVs are directly associated with SCTLD causation or progression remains unclear. Indeed, while the Peptostreptococcales-Tissierellales ASVs identified by Rosales et al. (2023) as potentially linked to SCTLD were not found linked to any other coral studies, a BLASTn analysis of our ASVs corresponding to this order identified overlaps with other coral diseases. For example, SCTLD bioindicator match ASVs ASV282 and ASV554 were 100% matches for the overlapping region of bacterial sequences previously detected in association with black band disease mats (Barneah et al., 2007).

The order Clostridiales has similarly been previously suggested to be linked to SCTLD (Meyer et al., 2019; Clark et al., 2021; Iwanowicz et al., 2020). Rosales et al. (2023) identified that this order was enriched in SCTLD lesions but was not found in acroporid corals experiencing an unrelated tissue loss disease. As acroporids are not thought to be affected by SCTLD (National Oceanic and Atmospheric Administration (NOAA), 2018), the authors concluded that this finding suggests Clostridiales enrichment may be particularly linked to SCTLD, rather than broadly linked to coral tissue loss diseases (Rosales et al., 2023). In our study, 30 ASVs representing 11,509 total reads were classified to the order Clostridiales, with the vast majority of these reads (11,386) sourced from diseased mesocosms. Just 96 total reads (spanning 8 ASVs) were associated with healthy mesocosms, 27 reads (all from ASV196) were associated with the FSW sample, and no Clostridiales reads were detected in any of the reagent controls. Similar to our Peptostreptococcales-Tissierellales ASVs, some, but not all, ASVs of the order Clostridiales were 100% sequence matches for the overlapping region of sequences previously identified as associated with other coral diseases (*e.g*., ASV982 to black band disease; Hadaidi et al., 2018). Thus, as with the bioindicators, whether any of these ASVs are directly associated with SCTLD causation or progression remains to be established.

## Conclusions

Here we investigated the size class of microorganisms(s) associated with transmission of SCTLD. Using concentrated water from mesocosms containing either diseased or healthy corals, we size fractioned the associated microbial community into their respective size classes using different sized filters: 0.8 µm pore size filters to capture microeukaryotes and expelled zooxanthellae, 0.22 µm pore size filters to capture bacteria and large viruses, and 0.025 µm pore size filters to capture small viruses. We also retained the liquid filtrate from the tangential flow filtration (TFF) microbial community concentration step to test for the effects of chemicals/toxins/toxicants <100 kDa in size. We then applied these filters and liquid TFF filtrate directly to healthy corals and monitored for disease onset. Using this approach over three separate experimental runs, we obtained mixed results that we have interpreted with caution, due to numerous potentially confounding factors. Following Run 1, microbes retained on the 0.22 µm pore size filters caused tissue loss in two healthy corals, suggesting that bacteria, large viruses, microeukaryotes, or infected zooxanthellae between 0.22 and 200 µm in size are more likely to be involved in transmission of the disease than larger (>200 µm) eukaryotes, small (<0.22 µm) viruses, or chemicals/toxins. However, the presence of *Vibrio corallilyticus* in both instances of apparent SCTLD transmission could represent a confounding factor. Run 2 experienced no transmission of disease, presumably due to the relatively low susceptibility of the receiver corals. Run 3 experienced apparent transmission across most treatment types, often including those treatments sourced from healthy donor corals, indicating that, although they appeared healthy, the receiver corals used were likely in a compromised health state due to a tissue loss outbreak among tankmates. We also characterized the prokaryotic community retained on the 0.22 µm pore size filters using Illumina sequencing of the V4 region of the 16S rRNA gene. We identified ASVs within our 0.22 µm pore size filter dataset that represented 100% identical matches for the overlapping region of sequences previously proposed as possible SCTLD bioindicators. In comparing against other 16S rRNA gene SCTLD datasets, we observed additional overlap with SCTLD-associated samples from studies spanning different geographic regions, years, source material (coral tissue/mucus, sediment, or biofilm), and coral species. This consistency suggests some of these bacteria, including potentially those of the orders Peptostreptococcales-Tissierellales and Clostridiales, may be associated with SCTLD progression.

## Additional Information and Declarations

### Field Study Permissions

The following information was supplied relating to field study approvals (*i.e*., approving body and any reference numbers): Field collections of coral colonies used in this experiment were conducted under permits FKNMS-2017-128-A2 and FKNMS-2019-160 from the Florida Keys National Marine Sanctuary. We also wish to thank Coral Restoration Foundation (Project IDs CRF-2019-011 (VJP) and CRF-2020-004 (CAK)) and Mote Marine Laboratory for providing the receiver corals used in the transmission experiments.

### Data Availability

The following information was supplied regarding data availability: The data is available *via* NCBI BioProject PRJNA918331 and USGS Data Release: Kellogg, C.A., Evans, J.S., and Voelschow, J.J., 2023, Prokaryotic communities shed by diseased and healthy corals (*Diploria labyrinthiformis*, *Pseudodiploria strigosa*, *Montastraea cavernosa*, *Colpophyllia natans*, and *Orbicella faveolata*) into filtered seawater mesocosms–raw and processed data: U.S. Geological Survey data release, https://doi.org/10.5066/P9LRIX9E.
